# Is this way; Self inflicted fracture of styloid process cures stylalgia

**Published:** 2013-06-25

**Authors:** A Kaur, A Singh, R Singal, S Gupta

**Affiliations:** * Department of Physiology, Maharishi Markandeshwer Institute of Medical Sciences and Research, Mullana, Ambala, India; ** Department Of Otolaryngology (E.N.T) M. M. Institute of Medical Sciences and Research, Mullana, Ambala, India; *** Department of Surgery, Maharishi Markandeshwer Institute of Medical Sciences and Research, Mullana, Ambala, India; **** Department of Radiodiagnosis and Imaging, Maharishi Markandeshwer Institute of Medical Sciences and Research, Mullana, Ambala, India

**Keywords:** Styloid syndrome, fracture, Stylalgia, ossification, Styloidectomy

## Abstract

The treatment of stylalgia varies from region to region. The initial treatment for stylalgia is conservative and if not relieved, styloidectomy is advised. Styloid process fracture has also given favorable results in many patients. We are presenting a rare case of a 45-year-old man who accidentally fractured his own styloid process and got relieved of stylalgia. According to our research, this is probably the first case in the world. In this case report, the authors also discuss the clinical presentation, differential diagnosis of stylalgia and various lines of management for stylalgia.

## Introduction

The styloid process is a cylindrical, long cartilaginous bone located on the posterior lower surface of the petrosal bone. The direction of this process is downwards to the front and slightly to the inside. The normal length of the styloid process is between 20-30 mm. It develops from the second brachial arch [**1**]. Patients can be categorized into two groups: a) who have classical symptoms of a foreign body sensation in the throat with a palpable mass in the tonsillar region b) those have pain in the neck following the carotid artery distribution (carotid artery distribution) [**2**]. The elongated styloid process can be palpated orally along the occlusal line in the posterior tonsillar fossa [**3**]. If palpation of the styloid process produces pain, which is referred to ear, head or face, it means that styloid process is elongated [**4**]. Initially, the clinician should try to decrease any muscle spasm and scar tissue around the styloid process or fracturing of styloid process with mixed results. Steroid injections have also been injected into the affected tissues with varying results [**5,6**]. If not relieved, then surgical excision of styloid process can be done. In 1949, Eagle described a syndrome as Eagles syndrome characterized by elongated styloid process or ossified stylohyoid ligament [**7**]. Here we report a case of a patient who accidentally fractured his styloid process, which cured his stylalgia.

## Case report

A 45-year-old man, farmer according to his occupation came with pain in the throat that has been lasting for 4 to 5 years. It was more during swallowing food and predominantly on the left side. With the time, symptoms aggravated and pain was radiating to the ear and sometimes to the ipsilateral face and rarely to the clavicle. Intraoral palpation revealed bilateral enlargement of styloid process. Thinking in terms of stylalgia, carbamazepine was started with a dosage of 200 mg twice a day. The patient was advised to come after 10 days for follow up. He did turn up but after around three months. As his symptoms were not relieved, he went to a quack, who rubbed salt like powder (locally called as manjun) in the region of palatine tonsils. 

**Fig. 1:**
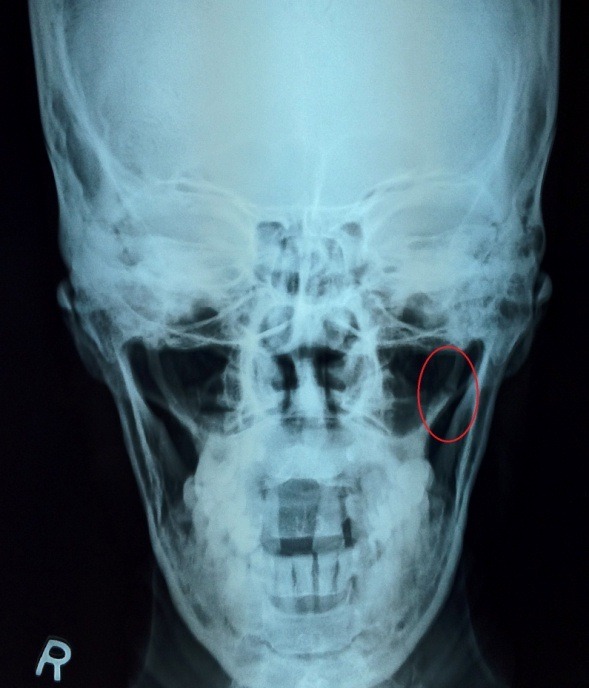
Red circle showing healed fracture of left styloid process

 As the patient describes, it gave him a moment of relief. The patient started rubbing the powder by himself in the tonsillar region for relief. Once while applying the powder he vigorously rubbed, pressed, and felt like something snapped. He felt severe excruciating pain, which subsided with painkillers in a week. In two weeks time he realized that he had no further pain and its radiation to the ear and other regions has also stopped. He presented to us to know whether he has caused himself an unknown trauma. Intraoral palpation still revealed bilateral enlargement of styloid process. X-ray of skull, Townes view was done. In addition to bilateral enlargement of styloid process it also showed a healed fracture of styloid process on the left side (**Fig. 1**).

## Discussion 

Normally, the styloid process is not palpable on bimanual palpation of tonsillar fossa and if palpable, it confirms the diagnosis. X-ray of skull Townes view can also be done to confirm the diagnosis. An ossified stylohyoid ligament or an elongated styloid process may not be symptomatic in every patient. Symptoms may vary from foreign body sensation, throat pain, dysphagia, headache, ipsilateral otalgia and sometimes facial and carotid pain [**8-10**]. In medical literature, there are not many reported cases of fractures ossified ligaments either these are spontaneous or traumatic [**10,11**]. Elongated styloid process can be caused by congenital elongation of styloid process due to the persistence of cartilaginous analog of the styloid and can also be due to calcification of stylohyoid ligament by unknown mechanism and growth of osseous tissue at the insertion of stylohyoid ligament. Glossopharyngeal nerve, vagus and 3rd branch of trigeminal nerve and chorda tympani can be stimulated by the styloid process and induce pain. There are various claims that fracture of styloid process can lead to granular tissue formation thus releasing pressure to nearby structures. Depending on the intensity of pain and dysphagia, treatment may vary from medical to surgical line of management. 

 The medical line includes anti-inflammatory and corticosteroid drugs. Surgery is planned in patients who do not respond to the medical line of management. Elongated styloid process can be excised, but some do not agree for excision of styloid process [5,6]. Excision of styloid process can be done by transoral approach or external approach. Nowadays, the styloid process is also resected endoscopically through transoral route [**12**]. The advantage of the following external approach is a proper exposure of styloid process and other vital structures [**13**]. 

 In spite of the medical research going all over the world and new drugs and treatment modalities coming every other day, few diseases still are not satisfactorily treated, neuralgia being one of them. These sequences of events highlights on three major issues. Firstly, to confirm the diagnosis of styloid process enlargement clinically by proper history and examination which should be further consolidated by X-ray Townes view. Secondly, the unsatisfactory treatment of stylalgia and associated neuralgias due to the lack of a set of guidelines for the treatment of the same (treatment ranges from medicines to styloidectomy to fracture of styloid process) and last but not the least the invasion of health industry in India by unqualified and under qualified practitioners. Our patient is relieved of symptoms and he is full of praise for the quack, but we as a medical fraternity should recognize this as a failure on our part.

## Conclusion 

The styloid process enlargement is not uncommon. History should be taken thoroughly and in detail and the physical examination of the head and neck are mandatory. Traditionally, the surgical excision of the styloid process is the treatment of choice. However, there have been cases all over the world in which the patient feel totally relieved of the neuralgic symptoms by infracture of styloid process after tonsillectomy. A patient infracturing a styloid process accidentally and getting relieved of stylalgia is rare and probably the first case in the world.
